# CAIC-Net: Robust Radio Modulation Classification via Unified Dynamic Cross-Attention and Cross-Signal-to-Noise Ratio Contrastive Learning

**DOI:** 10.3390/s26030756

**Published:** 2026-01-23

**Authors:** Teng Wu, Quan Zhu, Runze Mao, Changzhen Hu, Shengjun Wei

**Affiliations:** 1 School of Cyberspace Science and Technology, Beijing Institute of Technology, Beijing 100811, China; 3220205106@bit.edu.cn (T.W.); 3220245307@bit.edu.cn (Q.Z.); 3120205527@bit.edu.cn (R.M.); chzhoo@bit.edu.cn (C.H.); 2 Beijing Key Laboratory of Software Security Engineering Technology, Beijing Institute of Technology, Beijing 100811, China

**Keywords:** automatic modulation classification, deep learning techniques, feature-based extraction, dynamic cross-attention

## Abstract

In complex wireless communication environments, automatic modulation classification (AMC) faces two critical challenges: the lack of robustness under low-signal-to-noise ratio (SNR) conditions and the inefficiency of integrating multi-scale feature representations. To address these issues, this paper proposes CAIC-Net, a robust modulation classification network that integrates a dynamic cross-attention mechanism with a cross-SNR contrastive learning strategy. CAIC-Net employs a dual-stream feature extractor composed of ConvLSTM2D and Transformer blocks to capture local temporal dependencies and global contextual relationships, respectively. To enhance fusion effectiveness, we design a Dynamic Cross-Attention Unit (CAU) that enables deep bidirectional interaction between the two branches while incorporating an SNR-aware mechanism to adaptively adjust the fusion strategy under varying channel conditions. In addition, a Cross-SNR Contrastive Learning (CSCL) module is introduced as an auxiliary task, where positive and negative sample pairs are constructed across different SNR levels and optimized using InfoNCE loss. This design significantly strengthens the intrinsic noise-invariant properties of the learned representations. Extensive experiments conducted on two standard datasets demonstrate that CAIC-Net achieves competitive classification performance at moderate-to-high SNRs and exhibits clear advantages in extremely low-SNR scenarios, validating the effectiveness and strong generalization capability of the proposed approach.

## 1. Introduction

In modern wireless communication systems, automatic modulation classification (AMC) plays a pivotal role in enabling intelligent spectrum management, interference detection, and cognitive radio (CR). As communication environments become increasingly complex and spectrum resources continue to tighten, AMC has become indispensable for improving spectral efficiency and enhancing system autonomy [1]. Early AMC approaches relied heavily on manually engineered features—such as higher-order cumulants—and likelihood-based classifiers. However, as signal formats diversify and channel conditions grow more dynamic, these traditional methods exhibit clear limitations in both performance and generalizability [2].

In recent years, deep learning (DL) models such as convolutional neural networks (CNNs), recurrent neural networks (RNNs), and their variants have become the dominant paradigm in AMC research because of their strong automatic feature extraction capabilities [3]. These models can directly process raw in-phase/quadrature (I/Q) samples or constellation diagrams, eliminating the need for handcrafted features and achieving impressive classification accuracy under high-signal-to-noise ratio (SNR) conditions [4].

Despite these advances, deploying DL-based AMC models in real-world wireless environments remains challenging. During transmission, wireless signals are inevitably corrupted by additive white Gaussian noise, fading, and interference, often resulting in an extremely low SNR at the receiver [5]. Although existing DL models can achieve near-perfect accuracy at a high SNR, their performance degrades sharply when the SNR falls below 0 dB, as noise severely distorts the discriminative features required for classification [6]. Enhancing model robustness under low-SNR conditions is therefore a critical requirement for practical AMC applications [7]. Recognizing the complementary advantages of ConvLSTM [8] in extracting localized temporal dynamics and Transformer architectures [9] in modeling global long-range dependencies, recent studies have increasingly turned to hybrid dual-stream architectures. However, most existing hybrid models simply concatenate or statically combine features after extraction. Such static fusion strategies fail to enable deep, data-dependent bidirectional interaction between the two branches, resulting in redundant representations and suboptimal fusion efficiency. Consequently, the discriminative capability of these models remains limited under complex channel conditions.

To address these two fundamental challenges, this paper proposes CAIC-Net, a novel and robust radio modulation classification framework based on cross-attention–driven invariance learning. CAIC-Net introduces architectural innovations and optimized learning strategies to fundamentally improve low-SNR robustness and feature fusion efficiency. The model employs a dual-stream structure composed of ConvLSTM2D and Transformer blocks to jointly capture local temporal patterns and global dependencies. A Dynamic Cross-Attention Unit (CAU) is designed to facilitate deep bidirectional interaction between the two branches, while an SNR-aware mechanism adaptively adjusts the fusion strategy under varying channel conditions. Furthermore, a cross-SNR contrastive learning (CSCL) module is incorporated as an auxiliary task to enforce noise-invariant feature representations by constructing positive pairs across different SNR levels and optimizing them using InfoNCE loss.

The main contributions of this work are summarized as follows:

(1) We propose CAIC-Net, a dual-stream robust classification framework that integrates ConvLSTM2D-based local temporal modeling with Transformer-based global dependency modeling. The framework introduces fundamental improvements over traditional hybrid architectures in both structural design and learning strategy.

(2) We design a Dynamic Cross-Attention Unit (CAU) that enables deep bidirectional interaction between the two feature streams. By using one branch as the query and enhancing it through residual connections, CAU ensures effective feature complementarity and overcomes the limitations of simple concatenation, resulting in more discriminative fused representations.

(3) We introduce Cross-SNR Contrastive Learning (CSCL) as an auxiliary task. By constructing positive sample pairs across different SNR levels and optimizing them with InfoNCE loss, CSCL enforces intrinsic noise invariance in the learned representations, significantly improving robustness and generalization.

(4) We conduct extensive experiments on RadioML2016.10a and RadioML2016.10b datasets, demonstrating that CAIC-Net achieves strong robustness under low-SNR conditions and competitive performance at moderate-to-high SNRs. Notably, CAIC-Net achieves substantial performance gains under extremely low-SNR conditions (SNR < 0 dB), validating the effectiveness and robustness of the proposed approach.

## 2. Related Work and Motivation

### 2.1. Traditional Modulation Classification Methods

Traditional automatic modulation classification (AMC) approaches can be broadly categorized into two major paradigms: likelihood-based methods (LBMs) and feature-based methods (FBMs) [10]. Likelihood-based methods are rooted in optimal detection theory, where the core idea is to compute the posterior probability density of the received signal under different modulation hypotheses and make decisions according to the maximum likelihood criterion [11]. In theory, LBMs can achieve optimal classification performance under ideal conditions. However, their practical deployment faces two major challenges: first, channel parameters are difficult to estimate accurately in dynamic wireless environments [12]; second, evaluating the complex integrals involved in likelihood computation requires substantial computational resources, making LBMs unsuitable for real-time or resource-constrained applications [13].

Feature-based methods simplify the classification task by manually designing statistical features [14]. In recent years, several more advanced feature-based approaches have been proposed to improve the discriminative capability of handcrafted features. For example, phase diagram-based methods leverage nonlinear and nonparametric signal representations and have demonstrated promising performance when combined with machine learning techniques, such as phase diagram entropy-based modulation recognition in underwater acoustic and spread-spectrum communication scenarios [15]. In addition, compressive sensing-based feature extraction has been explored to reduce sampling complexity while preserving key signal characteristics, enabling effective modulation classification using compressed measurements and conventional classifiers [16].

Despite their improved performance compared to classical statistical features, these feature-based approaches still rely on carefully designed signal representations and domain-specific prior knowledge. More importantly, their robustness remains sensitive to severe noise and channel distortions, particularly in extremely low-SNR environments [17]. This limitation motivates the adoption of data-driven deep learning frameworks that can automatically learn noise-invariant representations directly from raw I/Q samples.

### 2.2. Deep Learning for AMC

In recent years, deep learning (DL) has rapidly emerged as the dominant approach for automatic modulation classification (AMC), owing to its powerful capabilities in automatic feature extraction and nonlinear representation learning. DL-based models can process raw I/Q sample sequences or derived representations like constellation diagrams and time-frequency maps, removing the need for labor-intensive handcrafted feature engineering in traditional methods [18].

From an architectural perspective, convolutional neural networks (CNNs) were among the earliest and most widely adopted models in AMC. CNNs excel at extracting multi-scale local features and spatial patterns from I/Q sequences or two-dimensional constellation images. To better capture the inherent temporal dependencies of wireless signals, recurrent neural networks (RNNs) and their variants, such as long short-term memory (LSTM) networks, have also been extensively explored, demonstrating strong capabilities in modeling long-range temporal correlations [19].

To further enhance performance and capture richer global contextual information, researchers have increasingly investigated hybrid network architectures. For example, West and O’Shea [9] proposed the CLDNN model by integrating CNN and RNN components, achieving an accuracy of 85% under high-SNR conditions. Subsequent studies refined this hybrid paradigm: Jiang et al. [20] combined CLDNN with LSTM, achieving 90.8% accuracy, while Zou et al. [21] incorporated attention mechanisms into CLDNN and similarly achieved around 90% accuracy at a high SNR. More recently, Transformer architectures and their core self-attention mechanisms have been introduced into AMC to effectively capture global contextual dependencies. For instance, Liang et al. [22] integrated ResNeXt with attention modules and achieved 90% accuracy under high-SNR conditions. These hybrid architectures aim to leverage the complementary strengths of different neural network families, and they have demonstrated the potential to achieve near-perfect classification accuracy in high-SNR scenarios.

### 2.3. Limitations of Existing Methods and Motivation

Although deep learning (DL) has significantly advanced automatic modulation classification (AMC), especially under high-SNR conditions, two major limitations still hinder its practical deployment. First, existing models lack robustness in low-SNR environments. When the SNR drops below 0 dB, Gaussian noise and channel fading severely distort the discriminative features learned by neural networks, causing a sharp decline in classification accuracy. Current approaches do not incorporate mechanisms that enforce noise-invariant feature representations, making them vulnerable in real-world communication scenarios.

Second, hybrid architectures that combine ConvLSTM and Transformer modules suffer from inefficient feature fusion. Most existing designs rely on simple concatenation or static weighted summation, which fails to enable deep, data-dependent interaction between the two branches. As a result, the fused representations often contain redundant information and insufficient complementarity, limiting their discriminative capability under complex channel conditions.

These limitations highlight the need for a new AMC framework that can achieve effective integration of local and global features while simultaneously enhancing the intrinsic noise robustness of learned representations. This motivates the development of a more adaptive and noise-invariant modulation classification system suitable for practical low-SNR environments.

## 3. Proposed CAIC-Net Model Architecture

### 3.1. Overall Architecture

**Figure 1:**
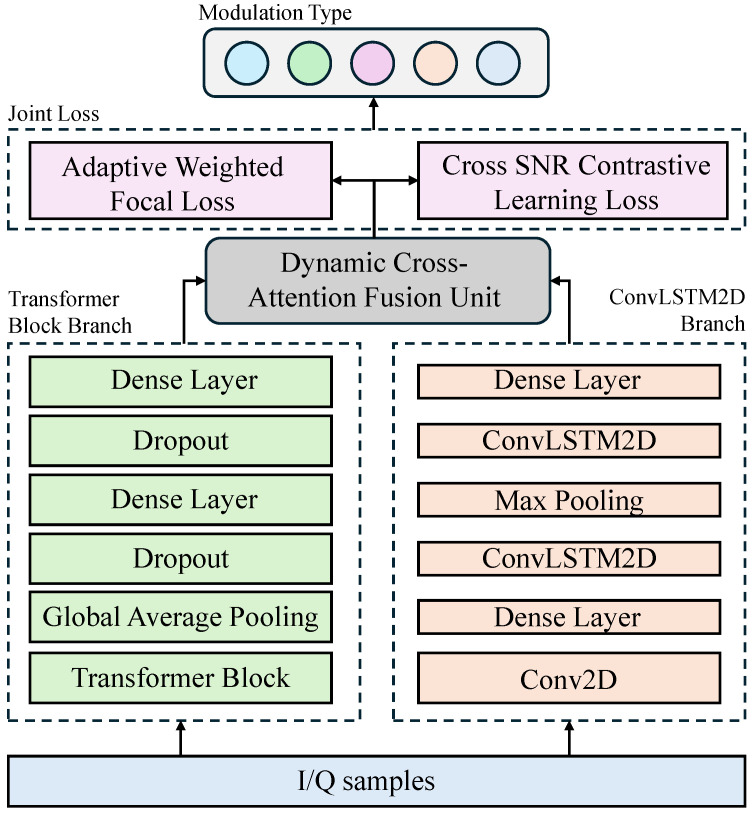
Overall Framework of CAIC-Net.

CAIC-Net adopts an end-to-end learning paradigm for radio modulation classification, and its overall architecture is illustrated in Figure 1. The central objective of the network is to construct a parallel dual-stream feature extraction module capable of efficiently capturing multi-dimensional discriminative representations from raw I/Q samples. The system consists of three key components: a dual-stream feature extractor, a Dynamic Cross-Attention Unit (CAU), and a dual-head output structure.

The input I/Q signal is simultaneously fed into two parallel feature extraction branches: a ConvLSTM2D branch and a Transformer Block branch. The ConvLSTM2D branch focuses on extracting local temporal features $F_{\mathrm{CL}}$, capturing short-term dynamics and spatiotemporal correlations. In contrast, the Transformer Block branch is designed to model long-range global dependencies $F_{T}$, capturing the overall structural and contextual relationships within the signal. This parallel design ensures that both local details and global contextual information are fully and independently encoded.

The outputs of the two branches are subsequently fused through the CAU, which serves as the core module for deep feature interaction. By employing a bidirectional cross-attention mechanism, the CAU enables mutual querying and enhancement between local and global features, effectively addressing the inefficiencies of static fusion strategies used in prior hybrid models. The resulting output is a residual-enhanced discriminative representation denoted as $F_{\mathrm{Final}}$.

Finally, the fused representation $F_{\mathrm{Final}}$ is forwarded to two separate output heads: a classification head for modulation prediction and a projection head for auxiliary constraints. The projection head maps $F_{\mathrm{Final}}$ into a low-dimensional embedding space used to compute the contrastive learning loss. The entire network is optimized using a joint loss function that combines primary classification loss with cross-SNR contrastive learning loss, thereby enforcing noise-invariant feature representations throughout the training process and significantly enhancing robustness under varying channel conditions.

### 3.2. Dual-Stream Feature Extraction

The core of CAIC-Net lies in its dual-stream feature extraction design, which is intended to overcome the limitations of single-architecture models in capturing the multi-dimensional characteristics of wireless signals. The received I/Q samples contain not only rich local temporal correlations and spatiotemporal patterns but also long-range global dependencies. To fully exploit these complementary properties, we construct two parallel branches: a ConvLSTM2D branch that focuses on fine-grained modeling of local spatiotemporal features and a Transformer Block branch that captures comprehensive global dependency structures. Working in tandem, these two branches provide a diverse and robust feature foundation for the subsequent dynamic cross-attention fusion.

#### 3.2.1. ConvLSTM2D Branch

Within the dual-stream feature extractor of CAIC-Net, the ConvLSTM2D branch is responsible for capturing local temporal dependencies and spatial structural patterns from the received I/Q signal sequences. RNNs often suffer from gradient vanishing when processing long sequences, whereas LSTM networks mitigate this issue through gated mechanisms. However, the gating operations in standard LSTM rely on fully connected layers, which limits their ability to preserve the spatial structure inherent in wireless signals. To address this limitation, ConvLSTM incorporates convolutional operations into the gating mechanism, enabling simultaneous modeling of temporal correlations and spatial locality, making it particularly suitable for two-dimensional representations of radio signals.

In a standard LSTM, information flow is regulated by three gates: the input gate, forget gate, and output gate. Their computations are defined as

(1) $$i_{t}=\sigma \;\left(W_{xi}X_{t}+W_{hi}H_{t-1}+W_{ci}C_{t-1}+b_{i}\right)$$

(2) $$f_{t}=\sigma \;\left(W_{xf}X_{t}+W_{hf}H_{t-1}+W_{cf}C_{t-1}+b_{f}\right)$$

(3) $$o_{t}=\sigma \;\left(W_{xo}X_{t}+W_{ho}H_{t-1}+W_{co}C_{t}+b_{o}\right)$$

where σ(·) denotes the sigmoid activation function, $X_{t}$ is the current input, $H_{t-1}$ is the previous hidden state, and $C_{t-1}$ is the previous cell state. $W_{x*}$, $W_{h*}$, and $W_{c*}$ denote the learnable parameters associated with the corresponding gate, representing the input-to-gate weights, hidden-to-gate weights, and peephole connections from the cell state, respectively, while $b_{*}$ denotes the bias term. The candidate cell state is computed as

(4) $${\tilde{C}}_{t}=\tanh\;\left(W_{xc}X_{t}+W_{hc}H_{t-1}+b_{c}\right)$$

and the updated cell state is obtained through:

(5) $$C_{t}=f_{t}⊙C_{t-1}+i_{t}⊙{\tilde{C}}_{t}$$

where the forget gate $f_{t}$ determines how much previous information is retained, and the input gate $i_{t}$ controls the incorporation of new information. The hidden state is then updated as

(6) $$H_{t}=o_{t}⊙\tanh\;\left(C_{t}\right)$$

This mechanism enables LSTM to preserve both short-term and long-term dependencies in sequential data.

**Figure 2:**
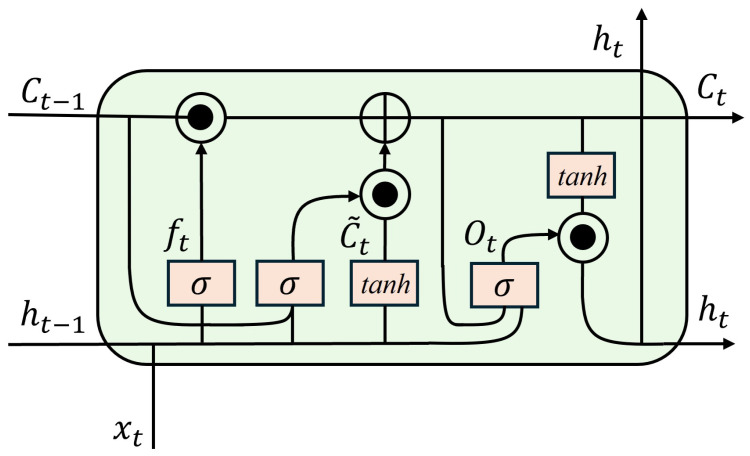
Architecture of the ConvLSTM module.

However, because standard LSTM uses fully connected operations, it cannot effectively capture spatial patterns in the input. As shown in Figure 2, ConvLSTM addresses this by replacing matrix multiplications with convolution operations in the gating equations:

(7) $$i_{t}=\sigma \;\left(W_{xi}X_{t}+W_{hi}H_{t-1}+W_{ci}⊙C_{t-1}+b_{i}\right)$$

(8) $$f_{t}=\sigma \;\left(W_{xf}X_{t}+W_{hf}H_{t-1}+W_{cf}⊙C_{t-1}+b_{f}\right)$$

(9) $$o_{t}=\sigma \;\left(W_{xo}X_{t}+W_{ho}H_{t-1}+W_{co}⊙C_{t}+b_{o}\right)$$

(10) $${\tilde{C}}_{t}=\tanh\;\left(W_{xc}X_{t}+W_{hc}H_{t-1}+b_{c}\right)$$

(11) $$C_{t}=f_{t}⊙C_{t-1}+i_{t}⊙{\tilde{C}}_{t}$$

(12) $$H_{t}=o_{t}⊙\tanh\;\left(C_{t}\right)$$

where ⊙ denotes element-wise multiplication. In ConvLSTM, the weight parameters $W_{x*}$, $W_{h*}$, and $W_{c*}$ correspond to learnable convolutional kernels applied to the input, hidden state, and cell state, respectively, replacing the fully connected transformations used in standard LSTM. These convolutional parameters are shared across time steps. By incorporating convolution into the gating mechanism, ConvLSTM preserves spatial locality while modeling temporal dynamics, enabling the extraction of more stable and noise resistant features under low-SNR conditions.

In CAIC Net, the ConvLSTM2D branch begins with convolution and pooling layers to perform initial feature extraction, reducing redundancy and highlighting local patterns. Stacked ConvLSTM layers then capture deeper temporal dependencies, while dropout is applied to prevent overfitting. The final hidden state produced by this branch serves as the local temporal feature representation, forming a strong foundation for the subsequent dynamic cross-attention fusion.

#### 3.2.2. Transformer Block Branch

In the dual-stream feature extractor of CAIC-Net, the Transformer Block branch is designed to model global dependencies within the received I/Q sequences, complementing the ConvLSTM2D branch, which focuses on local temporal and spatial patterns. Unlike RNN- or LSTM-based architectures that rely on recursive operations, the Transformer employs Multi-Head Self-Attention (MHSA) to perform information interaction across the entire sequence. This mechanism enables the model to explicitly capture long-range dependencies while suppressing noise-dominated, non-discriminative patterns—an ability that becomes particularly advantageous under low-SNR conditions.

To make the raw I/Q sequences compatible with the Transformer encoder, the input is first passed through a linear embedding layer and then augmented with positional encoding to preserve temporal ordering. The positional encoding is constructed using sinusoidal functions, allowing the model to distinguish relative positions across time steps.

In the *l*-th Transformer encoder layer, the input sequence $Z^{\left(l-1\right)}$ is linearly projected to generate the Query, Key, and Value matrices:

(13) $$Q=Z^{\left(l-1\right)}W_{Q},K=Z^{\left(l-1\right)}W_{K},V=Z^{\left(l-1\right)}W_{V}$$

where $W_{Q},W_{K},W_{V}\in R^{d\times d_{k}}$ are learnable parameter matrices, and $d_{k}$ denotes the dimensionality of the vectors for each attention head. The core attention computation is defined as

(14) $$\mathrm{Attention}\left(Q,K,V\right)=\mathrm{softmax}\;\left(\frac{QK^{⊤}}{\sqrt{d_{k}}}\right)V$$

where Attention(Q,K,V) denotes the attention output representation, obtained as a weighted aggregation of the value vectors *V* based on the similarity between queries and keys. This mechanism computes similarity between queries and keys to determine the relevance of different time steps and uses the resulting normalized attention weights to aggregate value vectors. In doing so, the model dynamically emphasizes signal segments most relevant to the current context while suppressing noise-dominated redundancy.

To further enhance representational capacity, the Transformer employs MHSA, which computes multiple attention heads in parallel across different subspaces:

(15) $$\mathrm{MHSA}\;\left(Z^{\left(l-1\right)}\right)=\left[\;\mathrm{Attention}\left(Q_{1},K_{1},V_{1}\right)\;∥\;\cdots \;∥\;\mathrm{Attention}\left(Q_{H},K_{H},V_{H}\right)\;\right]W_{O}$$

where *H* denotes the number of attention heads, ‖ represents concatenation, and $W_{O}\in R^{Hd_{k}\times d}$ is the output projection matrix. This multi-head design enables the model to capture diverse dependency patterns across multiple representational subspaces, thereby enriching the global feature representation.

The output of the attention module is then processed through residual connections and layer normalization to stabilize training, followed by a feed forward network (FFN) that applies nonlinear transformations and dimensional expansion. Stacking multiple such encoder layers allows the Transformer branch to progressively extract higher-level global representations from the input signal.

### 3.3. Dynamic Cross-Attention Fusion Unit

To achieve effective integration between the two heterogeneous feature branches in CAIC Net, we design a Dynamic Cross Attention Fusion Unit (CAU). This module addresses the limitations of traditional dual-stream architectures, which often suffer from inefficient information fusion and insufficient exploitation of feature complementarity. By employing a structured cross-attention mechanism, the CAU establishes a deep interaction pathway between the ConvLSTM2D and Transformer branches, while an SNR-aware strategy enables adaptive and robust fusion under varying channel conditions.

**Figure 3:**
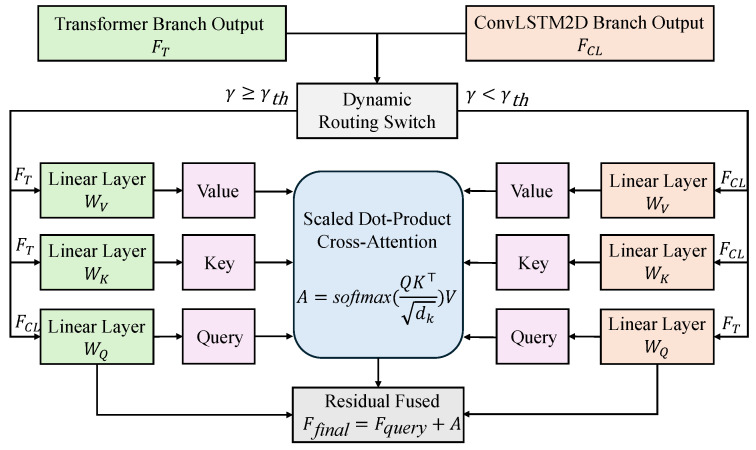
SNR-aware dynamic cross-attention fusion unit (CAU) in CAIC-Net.

As shown in Figure 3, the core of CAU is based on a standard cross-attention formulation, where the feature representation from one branch is used as the Query, and the features from the other branch serve as the Key and Value to provide complementary information. Let the ConvLSTM branch output be $F_{\mathrm{CL}}\in R^{T\times d}$ and the Transformer branch output be $F_{T}\in R^{T\times d}$. The general cross attention computation is expressed as

(16) $$Q=F_{\mathrm{query}}W_{Q},K=F_{\mathrm{context}}W_{K},V=F_{\mathrm{context}}W_{V}$$

(17) $$A=\mathrm{softmax}\;\left(\frac{QK^{⊤}}{\sqrt{d_{k}}}\right)V$$

where $F_{\mathrm{query}}$ and $F_{\mathrm{context}}$ denote the feature sources acting as the query and context providers, respectively, and $W_{Q},W_{K},W_{V}$ are learnable projection matrices. The resulting $A\in R^{T\times d}$ represents the enhanced cross-branch interaction features.

Unlike conventional static bidirectional cross-attention, the proposed CAU introduces a dynamic control mechanism guided by the signal-to-noise ratio (SNR). Let the instantaneous SNR of the input signal be denoted as γ. The system adaptively determines the assignment of Query and Context according to the value of γ:

(18) $$\phi \left(\gamma \right)=\left\{\begin{array}{cc} Q=F_{T}W_{Q},\;K=F_{\mathrm{CL}}W_{K},\;V=F_{\mathrm{CL}}W_{V}, & \gamma <\gamma_{\mathrm{th}}\\ Q=F_{\mathrm{CL}}W_{Q},\;K=F_{T}W_{K},\;V=F_{T}W_{V}, & \gamma \geq \gamma_{\mathrm{th}} \end{array}\right.$$

where $\gamma_{\mathrm{th}}$ denotes the SNR threshold, which can be empirically set or learned during training. When the input falls into a low-SNR regime, the local temporal features extracted by the ConvLSTM branch are easily corrupted by noise, thereby reducing their discriminative capability. In this case, the Transformer branch is automatically assigned as the Query, while the ConvLSTM branch serves as the Context, enabling global dependency features to actively “query” local details and enhance their representation. Conversely, under high-SNR conditions, the Transformer branch may capture redundant or overly smoothed information, whereas the ConvLSTM branch can reliably extract salient temporal patterns. The system then switches roles, assigning the ConvLSTM branch as the Query and the Transformer branch as the Context, thereby enriching global semantics.

After the cross-attention interaction, CAU enhances the Query branch through a residual connection:

(19) $$F_{\mathrm{final}}=F_{\mathrm{query}}+A$$

where $F_{\mathrm{query}}\in R^{T\times d}$ denotes the feature representation selected as the Query branch according to the SNR-aware routing strategy in Equation (18), and $A\in R^{T\times d}$ represents the corresponding cross-attention output obtained by attending to the Context branch. The residual addition preserves the original Query features while injecting complementary information from the Context branch.

It is important to emphasize that the final output of CAU is not a simple concatenation of the two branches. Instead, it is the residual-enhanced representation of the dynamically selected Query branch. In other words, when the SNR is low, the fused representation originates from the Transformer branch after residual enhancement; when the SNR is high, it originates from the ConvLSTM branch. Through this SNR-adaptive interaction and selection mechanism, CAU effectively adjusts the fusion strategy to different channel conditions, significantly improving the discriminative power and robustness of the learned features. The resulting fused representation is then forwarded to the subsequent classification module to complete the modulation recognition task.

### 3.4. Joint Loss Function

The joint loss function in CAIC-Net is designed to enhance both robustness and discriminative capability under complex channel conditions by combining a primary classification loss with a cross-SNR contrastive learning loss. This unified optimization objective ensures accurate modulation classification while enforcing consistency of feature representations across varying SNR levels. For the main classification task, we employ Adaptive Weighted Focal Loss, which reduces the influence of easily classified samples and directs the model’s attention toward more challenging ones—an essential property to improve performance in low-SNR environments. The adaptive weighting mechanism further mitigates the effects of class imbalance and noise interference while avoiding numerical instability. Formally, the loss is defined as

(20) $$L_{\mathrm{Focal}}=-\sum_{i=1}^{M}\omega_{i}\left({\left(1-y_{i}^{\prime }\right)}^{\lambda N_{i}}\;\left(\log y_{i}-\log y_{i}^{\prime }\right)\right)$$

where $y_{i}^{\prime }$ denotes the predicted probability, $y_{i}$ is the ground truth label, $\omega_{i}$ is the class weight, λ is the focusing parameter, and $N_{i}$ represents the difficulty coefficient of sample *i*.

To further enforce noise-invariant representations, we introduce Cross-SNR Contrastive Learning Loss (CSCL). During training, samples within each mini-batch are grouped according to their modulation types. For a given anchor sample $x_{i}$, a positive sample $x_{p}$ is defined as another sample of the same modulation type but captured at a different SNR, while samples belonging to other modulation categories within the same mini-batch are treated as negatives. The anchor and positive samples are randomly drawn independent signal instances from the same modulation class and do not share the same underlying transmitted message realization; they differ only in modulation label consistency and observed SNR.

In practice, the cross-SNR constraint is enforced through a balanced mini-batch sampling strategy. Specifically, each mini-batch is constructed to include samples of the same modulation type drawn from different SNR levels, ensuring that for a given anchor sample $x_{i}$ with SNR $\gamma_{i}$, a positive sample $x_{p}$ from the same class but with $\gamma_{p}\neq \gamma_{i}$ is available within the mini-batch.

The fused representation $F_{\mathrm{Final}}$ is passed through a nonlinear projection head to obtain an embedding *E*, which is then optimized using a batch-wise InfoNCE loss:

(21) $$L_{\mathrm{CL}}=-\log\frac{\exp\;\left(\mathrm{sim}(E_{i},E_{p})/\tau \right)}{\sum_{E_{j}\in B_{i}}\exp\;\left(\mathrm{sim}(E_{i},E_{j})/\tau \right)}$$

where $B_{i}$ denotes the set consisting of the positive sample $E_{p}$ and all negative samples $E_{n}$ within the same mini-batch, excluding the anchor $E_{i}$; sim(·) denotes cosine similarity; and τ is a temperature coefficient. By maximizing the similarity between anchor-positive pairs while suppressing similarity to samples from other modulation categories within the same mini-batch, CSCL encourages embeddings of the same modulation type to cluster tightly in the latent space, thereby achieving invariance across different SNR levels. The overall training objective is expressed as

(22) $$L_{\mathrm{Joint}}=L_{\mathrm{Focal}}+\alpha L_{\mathrm{CL}}$$

where α controls the relative contribution of the contrastive loss. Through this joint optimization strategy, CAIC Net maintains strong classification accuracy while significantly improving robustness and generalization across diverse SNR conditions.

## 4. Experiments and Analysis of Results

### 4.1. Experiment Setup

#### 4.1.1. Datasets and Implementation Details

This study employs two widely used public modulation classification datasets, RadioML2016.10a [23] and RadioML2016.10b [24], as the primary sources for experimental evaluation. These datasets are standard benchmarks for assessing the performance of wireless signal classification models. RadioML2016.10a contains 11 modulation types, with each sample represented as a 2×128 IQ sequence sampled at 1 MHz. The SNR ranges from −20 dB to 20 dB in steps of 2 dB, yielding a total of 220,000 samples, of which 176,000 are used for training and 44,000 for testing. In contrast, RadioML2016.10b includes 10 modulation types with the same 2×128 sample structure, but the dataset size is significantly larger, comprising 1,200,000 samples in total. Each sample spans 128 μs, and the SNR range is identical to that of RadioML2016.10a.

The datasets are constructed to be balanced across modulation categories and SNR levels; therefore, the adopted standard split preserves a stratified distribution with respect to both modulation type and SNR. All reported results are obtained by averaging the performance over multiple runs with different random seeds, ensuring robustness and reproducibility across all SNR conditions.

For all experiments, a single unified model is trained using the standard training/testing split of the datasets, where 80% of the samples are used for training, and the remaining 20% are used for testing. Samples from all available SNR levels are jointly used during training, and the model is not trained separately for each SNR. During evaluation, we follow the common AMC protocol and report per-SNR classification accuracy by grouping test samples according to their corresponding SNR values. This setting enables a fair and consistent assessment of the model’s robustness across different noise conditions using the same trained model.

In this work, automatic modulation classification is performed at the receiver side using baseband I/Q samples after downconversion. The experiments follow the standard RadioML benchmark setting, in which the received signals are generated using a predefined channel and impairment configuration. Specifically, different SNR conditions are realized by adjusting the power of additive white Gaussian noise (AWGN) during dataset generation. In addition to AWGN, the dataset generation process includes Rayleigh and Rician fading, center frequency offset (CFO), and sample rate offset (SRO). These impairments are inherited directly from the benchmark dataset rather than being explicitly modeled or tuned in this work. Accordingly, our evaluation focuses on robustness under this standardized impairment setting, enabling fair comparison with existing AMC methods.

Regarding model architecture, we construct a dual-branch feature extractor consisting of a ConvLSTM-based branch and a Transformer-block-based branch. The ConvLSTM branch begins with a Conv1D layer followed by a MaxPooling1D layer for temporal compression. Two stacked ConvLSTM layers are then used to capture local temporal dependencies, followed by dropout and a GlobalAveragePooling1D layer to regularize and aggregate features, producing a final 64-dimensional representation. The Transformer branch shares the same initial preprocessing layers but replaces the ConvLSTM units with Transformer blocks to model global dependencies. After identical pooling and dense layers, this branch also outputs a 64-dimensional feature vector. The detailed layer configurations are summarized in Table 1.

All experiments are conducted on an Ubuntu 22.04 LTS system using an NVIDIA RTX 3090 GPU for acceleration. The models are implemented in PyTorch 1.12.1 with Python 3.8.20. Training is performed using the AdamW optimizer with an initial learning rate of $1\times 10^{-4}$, a batch size of 256, and 300 training epochs. The weighting coefficient α in the joint loss function is set to 0.1. The SNR threshold is fixed at $\gamma_{\mathrm{th}}=0\;\mathrm{SNR}$.

#### 4.1.2. Baselines and Evaluation Metrics

To provide a comprehensive and fair comparison, this study selects several representative deep learning models from recent AMC research as baseline methods. These baselines span a diverse set of architectural paradigms, including hybrid CNN–RNN frameworks, attention-enhanced networks, multi-branch feature fusion models, and improved recurrent architectures, thereby reflecting the major trends in current modulation classification research. Among them, the R&CNN model integrates convolutional and recurrent neural networks to jointly capture spatial features and temporal dependencies, achieving high accuracy and real-time performance in challenging underwater acoustic channels [25]. The ASCLDNN model extends the CLDNN architecture by incorporating an attention mechanism that assigns greater weight to critical features, significantly improving recognition performance under low-SNR conditions [19]. The DMFF CNN model adopts a dual modal fusion strategy by converting raw signals into both GAF images and IQ sequences, extracting complementary features via ResNet50 and complex valued CNNs, and integrating them through a dual-representation fusion strategy, resulting in enhanced robustness in noisy environments [26]. The CNN GRU hybrid model extracts local spatial structures through convolutional layers and captures temporal correlations using GRU units, forming a lightweight and interpretable classification framework [27]. IRLNet employs a dual-path architecture combining improved residual stacks with LSTM layers to jointly learn shallow and deep features, achieving strong generalization across multiple datasets [13]. The GRU CNN fusion model leverages higher-order cumulants, instantaneous features, and cyclic spectrum representations, and it integrates CNN and GRU branches in parallel, maintaining high accuracy even under severe noise conditions [28]. Collectively, these models serve as diverse and competitive baselines for evaluating the performance of the proposed CAIC Net.

For evaluation metrics, we adopt classification accuracy (AC), floating point operations per second (FLOPS), and memory usage as the primary indicators. Accuracy reflects the overall correctness of model predictions, while FLOPS and memory usage provide insights into computational efficiency and resource requirements. All models are evaluated using identical data splits, training configurations, and testing procedures to ensure fairness and consistency in comparison.

### 4.2. Overall Performance Comparison

Table 2 summarizes the overall performance of the proposed CAIC Net and several representative baseline models on the RadioML2016.10a dataset. The table reports classification accuracy at two representative SNR levels—0 dB and −16 dB—highlighting model behavior under moderate/high-SNR and extremely low-SNR conditions.

The results show that traditional hybrid architectures such as R&CNN achieve excellent performance at high SNR, reaching 99.45% accuracy at 0 dB. However, their performance collapses under severe noise, dropping to below 10% at −16 dB, indicating high vulnerability to noise corruption. Models enhanced with attention mechanisms or feature fusion strategy, such as ASCLDNN and DMFF CNN, maintain around 90% accuracy at a moderate SNR but similarly degrade to below 10% at −16 dB. These observations suggest that although attention and feature fusion strategies can strengthen feature representation, they remain insufficient to counteract extreme noise interference.

Among recurrent based models, the CNN-GRU and CNN GRU parallel architectures achieve 90% and 86% accuracy at 0 dB, respectively, but their performance drops sharply to below 20% and 55% at −16 dB. IRLNet performs strongly at a high SNR, achieving 92.4% accuracy at 0 dB, yet still suffers a substantial decline under low-SNR conditions, maintaining less than 50% accuracy at −16 dB. These results indicate that although recurrent structures and residual pathways help capture temporal dependencies and deep features, their robustness remains limited in extremely noisy environments.

In contrast, the proposed CAIC Net demonstrates clear advantages across both SNR levels. At 0 dB, CAIC Net achieves 92% accuracy, comparable to the best-performing baseline models. More importantly, at −16 dB, CAIC Net maintains a significantly higher accuracy of 64%, outperforming all competing methods by a large margin.

To verify performance stability under extreme noise, we further evaluated CAIC-Net at −16 dB using three different random seeds and 10-fold cross-validation. The resulting accuracy is 64.0% ± 1.8%, indicating that the reported performance is stable rather than incidental.

This substantial improvement is primarily attributed to the dynamic cross-attention fusion mechanism and the SNR-aware strategy, which enable the model to adaptively strengthen critical feature representations under noise-dominated conditions, thereby enhancing discriminative capability in low-SNR environments.

**Figure 4:**
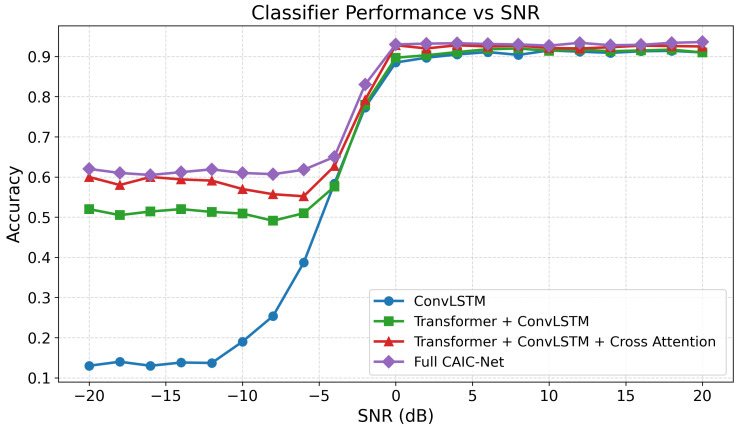
Classifier performance comparison.

Figure 4 illustrates the modulation classification accuracy of four models across different SNR levels on the RadioML2016.10b dataset. As the SNR increases, all models exhibit an upward performance trend; however, their behaviors diverge significantly in the low-SNR range.

The baseline ConvLSTM model performs poorly under low-SNR conditions and only approaches saturation at a high SNR, reflecting its sensitivity to noise. Introducing a Transformer branch (Transformer + ConvLSTM) improves performance in the mid-to-low SNR range, confirming the benefit of global dependency modeling for noise resilience. Incorporating cross-attention (Transformer + ConvLSTM + Cross-Attention) further stabilizes performance between −10 dB and 4 dB, with accuracy consistently surpassing the previous two models, demonstrating the effectiveness of cross-attention in feature fusion.

The full CAIC-Net model demonstrates the most pronounced performance advantages in the low-SNR regime, with particularly notable gains between −16 dB and 0 dB. Specifically, it reaches 92% accuracy at 0 dB and maintains over 60% accuracy at −16 dB, highlighting its strong robustness and discriminative capability under severe-noise conditions. At moderate and high SNR levels, CAIC-Net remains competitive with established hybrid architectures.

### 4.3. Sensitivity Analysis of the SNR Threshold

The proposed CAIC-Net introduces an SNR-aware feature interaction mechanism controlled by a threshold parameter $\gamma_{\mathrm{th}}$, which governs how the model adaptively adjusts its feature fusion behavior under different channel conditions. Rather than activating or deactivating a specific sub-module, $\gamma_{\mathrm{th}}$ serves as a global control variable that influences the relative emphasis of complementary feature representations across SNR regimes. Although $\gamma_{\mathrm{th}}$ is fixed to 0 dB in the default configuration, it is important to examine whether the overall performance is sensitive to this choice and to justify the rationality of selecting 0 dB as the switching point.

To this end, we conduct a sensitivity analysis by varying $\gamma_{\mathrm{th}}$ over a broad range from −10 dB to 10 dB using several discrete settings while keeping all other training and evaluation conditions unchanged. Following the standard automatic modulation classification (AMC) evaluation protocol, we report per-SNR classification accuracy at three representative operating points: an extreme low-SNR condition (−16 dB), a moderate-SNR condition (0 dB), and a high-SNR condition (10 dB).

**Figure 5:**
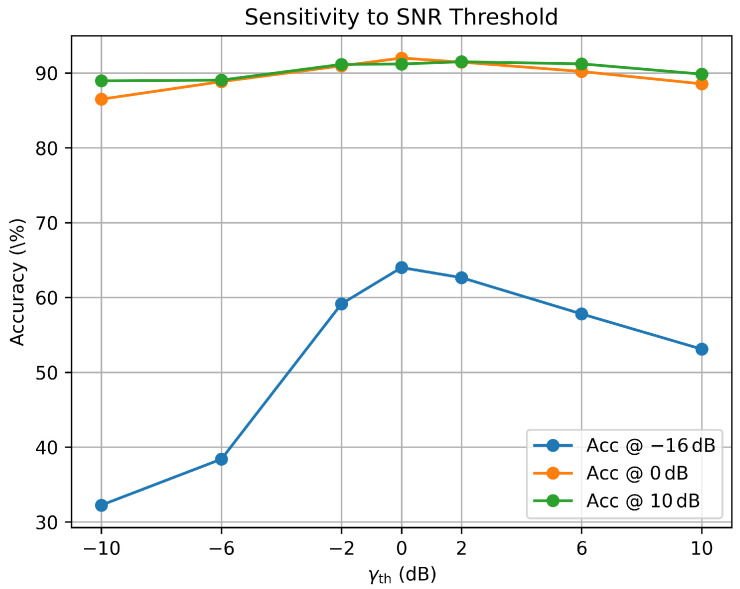
Sensitivity analysis of the SNR threshold $\gamma_{\mathrm{th}}$ on RadioML2016.10a.

As shown in Figure 5, the choice of $\gamma_{\mathrm{th}}$ has a pronounced impact on performance under extreme noise. In particular, the accuracy at −16 dB increases significantly as $\gamma_{\mathrm{th}}$ approaches 0 dB, reaching the peak value of 64.0% at $\gamma_{\mathrm{th}}=0$ dB, and then gradually decreases when $\gamma_{\mathrm{th}}$ is further increased. This trend is consistent with the design motivation of CAU: in the low-SNR regime, local temporal cues extracted by the ConvLSTM branch are more vulnerable to severe noise corruption, and a threshold around 0 dB allows the model to more frequently rely on globally consistent dependencies to enhance robustness. When $\gamma_{\mathrm{th}}$ is set too low, the switching mechanism is biased toward ConvLSTM-dominant querying even under extremely noisy inputs, leading to substantial degradation at −16 dB. Conversely, when $\gamma_{\mathrm{th}}$ is set too high, the querying role becomes overly biased toward the Transformer branch across a broader SNR range, which may suppress useful local temporal details and thus reduces the low-SNR benefit.

Meanwhile, the accuracies at 0 dB and 10 dB remain relatively stable across different $\gamma_{\mathrm{th}}$ values. This indicates that the routing threshold mainly affects the extremely low-SNR regime, while the model maintains competitive performance under moderate- and high-SNR conditions. Overall, $\gamma_{\mathrm{th}}=0$ dB provides the best trade-off in our setting: it yields the strongest robustness improvement under severe noise while preserving stable performance at moderate-to-high SNRs. Therefore, we adopt $\gamma_{\mathrm{th}}=0$ dB as the default threshold in the remainder of this work.

### 4.4. Ablation Study Analysis

To evaluate the contribution of each key component in CAIC-Net, we conduct four groups of ablation experiments focusing on three aspects: branch architecture, feature fusion mechanism, and loss function design. The detailed results are presented in Table 3.

From the perspective of branch architecture, removing either the ConvLSTM branch or the Transformer branch leads to a noticeable performance drop, though the degree of degradation differs. Eliminating the ConvLSTM branch reduces accuracy from 94% to 90% at 0 dB and from 64% to 55% at −16 dB, indicating that local temporal features play a crucial role in maintaining robustness under low-SNR conditions. In comparison, removing the Transformer branch results in accuracies of 89% at 0 dB and 52% at −16 dB, demonstrating that global dependency modeling is also indispensable, though its impact under an extremely low SNR is slightly weaker than that of the temporal branch. Together, these findings validate the complementary nature of the dual-branch design: the ConvLSTM branch excels at capturing local temporal patterns, while the Transformer branch models long-range dependencies, and only their joint operation yields stable and robust feature representations.

Regarding the feature fusion mechanism, removing the cross-attention fusion unit and replacing it with simple concatenation leads to a more substantial performance decline, especially under low-SNR conditions, where accuracy drops from 64% to 43%. This result highlights that dynamic cross-attention not only integrates complementary information from both branches but also adaptively adjusts the roles of Query and Context based on SNR, enabling the model to emphasize critical features and suppress noise-dominated interference. In contrast, static fusion methods cannot adapt feature weighting to varying channel conditions, resulting in significantly weaker performance under extreme noise.

In terms of loss function design, removing the Cross-SNR Contrastive Learning (CSCL) loss causes only a slight decrease in accuracy at 0 dB (from 92% to 90%) but leads to a substantial drop from 64% to 48% at −16 dB. This clearly demonstrates that CSCL primarily enhances feature consistency across different SNR levels. By constructing positive and negative sample pairs across SNR conditions and optimizing with InfoNCE loss, the model learns to pull together features of the same modulation type in the embedding space, thereby significantly improving discriminative capability under low-SNR conditions.

### 4.5. Parameter Efficiency Analysis

To further evaluate the computational efficiency of the proposed model, we compare several architectures in terms of trainable parameters, floating point operations (FLOPs), and memory usage. The results are presented in Table 4. This experiment aims to assess whether the model achieves higher parameter efficiency and deployment feasibility while maintaining strong recognition performance.

The results show that although the traditional CNN model performs reliably in modulation classification tasks, it contains as many as 5.45 million parameters, requires over 80 million FLOPs, and occupies 61.4 MB of memory, making deployment on resource constrained edge devices challenging. In contrast, the LSTM model significantly reduces both parameter count and memory footprint to 0.20 million and 2.31 MB, respectively, but its limited representational capacity makes it insufficient for capturing complex modulation patterns.

CAIC Net achieves a well-balanced parameter scale, with a total of 2.00 million parameters, 65.6 million FLOPs, and 23.6 MB of memory usage. These results indicate that CAIC-Net is suitable for deployment on resource-limited devices, where only inference is performed after offline training.

Beyond architectural and parameter-level comparisons, we further evaluate the training and inference efficiency of each model, as shown in Table 5. This experiment evaluates the computational efficiency of different models by separately reporting offline training cost and online inference latency, which are relevant to different stages of practical deployment.

The results indicate that the traditional CNN model is highly efficient in training, requiring only 30 s per epoch, with an inference time of 1000 μs per sample and a total training time of 600 s. However, its limited ability to capture temporal dependencies restricts its recognition performance. The LSTM model, while strong in temporal modeling, incurs a substantial computational cost, requiring 800 s per epoch and 44,800 s in total, with a slow inference speed of 2000 μs per sample, making it unsuitable for real-time applications.

The SCRNN model offers a more balanced trade off, with 280 s per epoch, 600 μs inference time, and a total training time of 11,480 s. ResNet, as a representative deep convolutional architecture, requires 341 s per epoch but achieves the fastest inference speed at only 183 μs per sample, demonstrating strong real-time capability. CAIC-Net incurs higher offline training costs than a standard CNN due to its dual-branch architecture but maintains a moderate training time compared with other temporal models.It requires 334 s per epoch, 2650 s in total, and 780 μs per sample for inference. While maintaining high recognition accuracy, CAIC-Net significantly reduces training cost and inference latency, demonstrating strong engineering practicality and deployment potential.

### 4.6. Robustness and Feature Visualization

#### 4.6.1. Confusion Matrix Analysis

**Figure 6:**
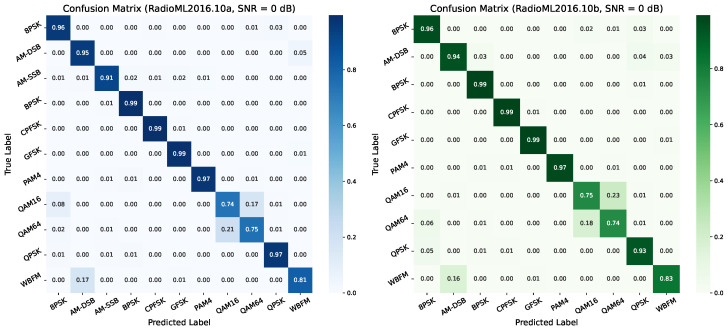
Confusion matrix at 0 dB SNR.

To further evaluate the modulation recognition performance of the proposed model under low-signal-to-noise ratio (SNR) conditions, Figure 6 presents the normalized confusion matrices on the RadioML2016.10a and RadioML2016.10b datasets at SNR = 0 dB. Each matrix uses true modulation labels as the vertical axis and predicted labels as the horizontal axis, with values indicating the classification proportion for each modulation type. These matrices reveal the model’s recognition accuracy and confusion patterns across different modulation categories.

On the RadioML2016.10a dataset, the model demonstrates high classification accuracy for most digital modulation types. In particular, BPSK, QPSK, GFSK, and CPFSK exhibit near-perfect recognition, with diagonal values exceeding 0.96. For analog modulations such as AM-DSB and AM-SSB, the model also performs well, achieving accuracies of 0.94 and 0.91, respectively. However, recognition accuracy drops for higher-order modulations like QAM16 and QAM64, both around 0.75, indicating that these complex constellation structures are more susceptible to noise-induced misclassification, even at moderate SNR levels.

In comparison, the confusion matrix for RadioML2016.10b shows a similar overall trend, with slight improvements in certain categories. BPSK, QPSK, GFSK, and CPFSK continue to maintain high recognition rates (≥0.96), while QAM16 and QAM64 reach 0.74, slightly higher than in the 10a dataset. Notably, WBFM achieves an accuracy of 0.83, outperforming its counterpart in the 10a dataset. These results suggest that the model generalizes well across datasets, especially for low-order digital and analog modulations, while higher-order modulations remain the primary source of classification errors.

**Figure 7:**
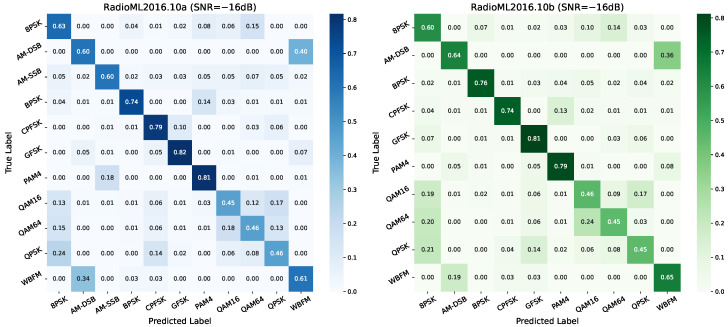
Confusion matrix at −16 dB SNR.

Under extremely low-SNR conditions (SNR = −16 dB), modulation recognition becomes significantly more challenging. Figure 7 presents the normalized confusion matrices for the RadioML2016.10a and RadioML2016.10b datasets at this noise level, illustrating the model’s classification performance and confusion behavior across modulation types.

Despite the severe noise interference, the model retains relatively high recognition accuracy for several modulation types in the RadioML2016.10a dataset. Digital modulations such as BPSK, QPSK, GFSK, and CPFSK show strong diagonal dominance, with most errors occurring among similar or closely related modulations. This indicates that these schemes preserve distinctive features even under harsh channel conditions. Analog modulations, including AM-DSB, AM-SSB, and WBFM, also exhibit stable performance, although some inter-class confusion is observed. In contrast, higher-order modulations such as PAM4, QAM16, and QAM64 display more dispersed diagonal energy and are frequently misclassified into other complex or structurally similar categories, highlighting their vulnerability to noise at an extremely low SNR.

The confusion matrix for RadioML2016.10b follows a similar pattern, showing strong recognition for low-order digital and analog modulations, while higher-order types remain prone to confusion. Notably, some digital modulations in the 10b dataset exhibit a more concentrated diagonal distributions, suggesting more stable feature representations. However, misclassification among higher-order modulations and between structurally similar types remains prominent. Overall, these results confirm that the proposed model maintains robust and generalizable performance for low-order and analog modulations under extreme noise, while high-order schemes continue to pose challenges for reliable classification.

#### 4.6.2. Feature Space Visualization

**Figure 8:**
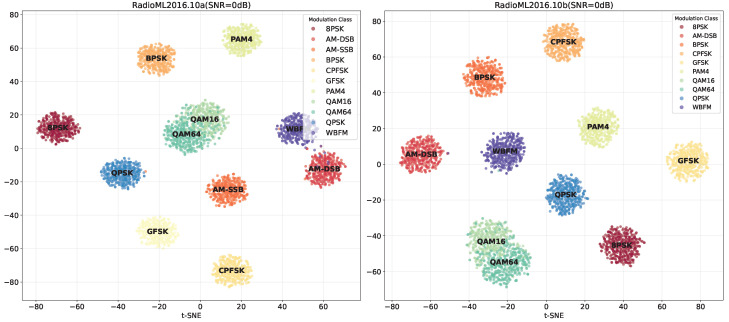
t-SNE visualization of the learned feature space at 0 dB SNR. Each point represents a sample, colored by modulation type. The axes denote the two-dimensional t-SNE embedding.

To further investigate the model’s discriminative capability across different modulation types and its structural behavior in the feature space, we employ t-SNE to visualize the dimensionality-reduced feature representations of the RadioML2016.10a and RadioML2016.10b datasets under SNR = 0 dB. Figure 8 illustrates the clustering patterns of modulation categories in a two-dimensional feature space obtained via t-SNE, where each point represents a sample and colors correspond to different modulation labels. The horizontal and vertical axes correspond to the two dimensions of t-SNE embedding and do not have explicit physical interpretations.

As seen in the visualization, it is evident that low-order-modulation schemes such as BPSK, QPSK, GFSK, and CPFSK form compact and well-separated clusters, indicating strong feature separability and effective representation learning for these categories. In contrast, higher-order modulations like QAM16 and QAM64 exhibit more ambiguous boundaries and noticeable overlap, suggesting that the model’s ability to distinguish complex constellation structures remains limited. Additionally, analog modulations such as AM DSB and WBFM show partial interclass overlap, implying potential instability in feature expression when handling analog signals, although the overall performance remains acceptable.

Notably, the RadioML2016.10a dataset includes the AM SSB category, which appears more dispersed in the feature space and overlaps with AM DSB, further confirming the difficulty of distinguishing analog modulations under moderate-SNR conditions. In comparison, the RadioML2016.10b dataset—featuring fewer modulation categories—exhibits a more compact clustering structure overall. However, confusion within the QAM family remains prominent, indicating that high-order modulations continue to pose challenges for reliable separation in the feature space.

## 5. Conclusions

In this work, we propose CAIC Net, a robust modulation classification framework tailored for low-SNR wireless communication environments. By constructing a dual stream feature extractor composed of ConvLSTM2D and Transformer branches, the model effectively integrates local temporal patterns with global dependency structures. To address the inefficiencies of traditional hybrid architectures in feature fusion, we design a Dynamic Cross-Attention Unit (CAU) that enables deep bidirectional interaction and residual enhancement between the two branches, resulting in more discriminative fused representations. Furthermore, to improve generalization under complex channel conditions, we introduce Cross-SNR Contrastive Learning (CSCL) as an auxiliary task. By minimizing the feature distance between samples of the same modulation type across different noise levels, CSCL significantly enhances the model’s intrinsic invariance to channel noise.

Experimental results on two benchmark datasets demonstrate that CAIC-Net achieves robust and reliable classification performance under challenging low-SNR conditions while maintaining competitive accuracy at moderate and high SNRs. These results confirm the effectiveness of the proposed SNR-adaptive fusion mechanism in practical wireless communication scenarios. Future work may explore the scalability of the CAU module to more general cross-representation signal fusion tasks, as well as the transferability of the CSCL strategy to other wireless learning problems, paving the way for broader deployment of intelligent modulation recognition systems in real-world communication environments. 

## Figures and Tables

**Table 1:** Model Layer Dimensions.

Layer Type	ConvLSTM Branch Output Shape	Transformer Branch Output Shape
Input	128×2	128×2
Conv1D	121×64	121×64
MaxPooling	60×64	60×64
ConvLSTM	60×64	60×64
Dropout	60×64	60×64
ConvLSTM/Transformer	60×64	60×64
Dropout	64	64
GlobalAveragePooling1D	64	64
Dense	20	20
Dropout	20	20

**Table 2:** Overall performance comparison on the RadioML2016.10a dataset.

Model	Input Signal	Architecture	Accuracy @ 0 dB	Accuracy @ −16 dB
R&CNN [25]	Sampled signal	CNN + GRU	99.45%	<10%
ASCLDNN [19]	IQ sequence	Attention + LSTM	90%	<10%
DMFF-CNN [26]	IQ sequence	DMFF + CNN	90%	<10%
CNN-GRU Model [27]	IQ sequence	CNN + GRU	90%	<20%
IRLNet [13]	IQ sequence	IRS + LSTM	92.4%	<50%
CNN-GRU Parallel Model [28]	IQ sequence	CNN + GRU	86%	<55%
CAIC-Net	IQ sequence	Transformer-block + ConvLSTM + cross-attention	92%	64%

**Table 3:** Ablation study comparison.

Model Variant	ConvLSTM	Transformer	Fusion	CSCL	Acc @ 0 dB	Acc @ −16 dB
CAIC-Net	✔	✔	✔	✔	92%	64%
w/o ConvLSTM	×	✔	✔	✔	90%	55%
w/o Transformer	✔	×	✔	✔	89%	52%
w/o Fusion	✔	✔	×	✔	87%	43%
w/o CSCL	✔	✔	✔	×	90%	48%

**Table 4:** Comparison of model parameter size and computational resources.

Model Architecture	Trainable Parameters	FLOPs	Memory Usage
CNN	5,456,219	80,548,043	61.4 MB
LSTM	199,563	7,696,283	2.31 MB
CAIC-Net	2,001,907	65,592,761	23.6 MB

**Table 5:** Comparison of training and inference time.

Model Architecture	Training Time per Epoch (s)	Inference Time (μs/sample)	Total Training Time (s)
CNN	30	1000	600
LSTM	800	2000	44,800
SCRNN	280	600	11,480
ResNet	341	183	1850
CAIC-Net	334	780	2650

## Data Availability

The raw data supporting the conclusions of this article will be made available by the authors on request.
